# Cheoplasty

**Published:** 1859-10

**Authors:** A. A. Blandy


### ARTICLE VI.

### *Cheoplasty.*

Messrs. Editors:

Having been absent from the United States during the past year, my interests have no doubt suffered as much from the consequent inattention, as from the efforts of those who have so frequently endeavored to misrepresent this process.

Since that time I have made many improvements, especially in the metal, which is now capable of running clasps in such pieces when they become necessary. Also, in many features of its manipulation, producing more accuracy and saving of time.

We have in test a new process of plating or galvanising, which promises to give results much more reliable than our former methods, originating, I am informed, with Dr. Thompson, of Glasgow, a gentleman highly skilled in the working of batteries, and who is using Cheoplasty in all cases plated, thus making it one of the most beautiful products of all mechanical work. He has kindly promised a paper upon this subject.

I have also noticed, during my absence, statements made by certain parties which are totally untrue, in relation to its going down, &c., and I beg to state that those entrusted with its management have been constantly making sales of rights, and I presume that at least 600 pounds of metal have been sold during the past year, and since my return I have been compelled to manufacture a large stock to supply demands. This will establish the facts better than any amount of words or other statements.

My process is still the most economical, speediest, most reliable, least likely to break, and the most readily repaired of any yet known, and is so endorsed by hundreds of the best men in the profession at this time. And I claim to be familiar with all processes in their exact detail.

In conclusion, allow me to state, that it is my intention, the earliest moment possible, to place in the hands of my lawyers, my tooth-patents so very generally infringed upon by users and by manufacturers, and now being used by several other processes. This statement is due from me to those who may possibly think, from my absence, that I have abandoned claims undoubtedly secured to me by government, and which have cost me much money, time and labor, and which I will protect, cost what it may, as soon as it is possible for me to do so.

A. A. BLANDY, M. D., &c.

